# The Identification of a Novel Gene, *MAPO2*, That Is Involved in the Induction of Apoptosis Triggered by O^6^-Methylguanine

**DOI:** 10.1371/journal.pone.0044817

**Published:** 2012-09-24

**Authors:** Ryosuke Fujikane, Masayuki Sanada, Mutsuo Sekiguchi, Masumi Hidaka

**Affiliations:** 1 Department of Physiological Science and Molecular Biology, Fukuoka Dental College, Fukuoka, Japan; 2 Advanced Science Research Center, Fukuoka Dental College, Fukuoka, Japan; German Cancer Research Center, Germany

## Abstract

O^6^-Methylguanine, one of alkylated DNA bases, is especially mutagenic. Cells containing this lesion are eliminated by induction of apoptosis, associated with the function of mismatch repair (MMR) proteins. A retrovirus-mediated gene-trap mutagenesis was used to isolate new genes related to the induction of apoptosis, triggered by the treatment with an alkylating agent, *N*-methyl-*N*-nitrosourea (MNU). This report describes the identification of a novel gene, *MAPO2* (O^6^-methylguanine-induced apoptosis 2), which is originally annotated as *C1orf201*. The *MAPO2* gene is conserved among a wide variety of multicellular organisms and encodes a protein containing characteristic PxPxxY repeats. To elucidate the function of the gene product in the apoptosis pathway, a human cell line derived from HeLa MR cells, in which the *MAPO2* gene was stably knocked down by expressing specific miRNA, was constructed. The knockdown cells grew at the same rate as HeLa MR, thus indicating that MAPO2 played no role in the cellular growth. After exposure to MNU, HeLa MR cells and the knockdown cells underwent cell cycle arrest at G_2_/M phase, however, the production of the sub-G_1_ population in the knockdown cells was significantly suppressed in comparison to that in HeLa MR cells. Moreover, the activation of BAK and caspase-3, and depolarization of mitochondrial membrane, hallmarks for the induction of apoptosis, were also suppressed in the knockdown cells. These results suggest that the *MAPO2* gene product might positively contribute to the induction of apoptosis triggered by O^6^-methylguanine.

## Introduction

S_N_1 type alkylating agents, such as *N*-methyl-*N*-nitrosourea (MNU) and *N*-methyl-*N′*-nitro-*N*-nitorosoguanidine (MNNG), produce various methylated bases, most of which are believed to be efficiently repaired by base excision repair (BER) [1], [2]. O^6^-methylguanine is especially important, since it allows the progression of replication fork and can mispair with thymine during DNA replication [3], [4], [5], thus leading to a GC to AT transition. To preserve genome integrity from the mutagenic insult, organisms possess a specific repair protein, O^6^-methylguanine-DNA methyltransferase (MGMT), which transfers a methyl group from O^6^-methylguanine to a methyl-acceptor cysteine residue in the molecule [6], [7], [8]. O^6^-methylguanine that is not repaired by MGMT has chance to pair with thymine, and such a mispair is recognized by a mismatch repair (MMR) protein complex, composed of MSH2, MSH6, MLH1 and PMS2 [9], [10], [11]. Thereafter, a series of proteins are activated and a signal is delivered to induce apoptosis [12]. Reflecting the distinct roles of MGMT and MMR proteins, *Mgmt^−/−^* mice are hypersensitive to the killing effect of alkylating agents and display a decrease in size of the thymus and hypocellular bone marrow after MNU administration [13], [14], [15]. Furthermore, mice with mutations in both the *Mgmt* gene and one of the mismatch repair genes, such as *Mlh1*, are as resistant to MNU as are wild-type mice, in terms of survival, but do develop numerous tumors after receiving MNU [16]. These findings clearly indicate that the MMR proteins-dependent apoptosis contributes to the suppression of cells predisposed to form tumors [16], [17].

Following the recognition of O^6^-methylguanine-thymine mispairs by MMR proteins, ATR kinase is activated, which in turn phosphorylates CHK1 protein, thus leading to the activation of a cell cycle checkpoint [18]. The depolarization of mitochondrial membranes followed by the activation of caspase-3 is induced, through the regulation of the activities of the BCL-2 family of proteins [19], [20]. However, the precise molecular mechanism that activates the signaling cascade leading to apoptosis has been elusive.

The gene-trap method is one of the elaborate techniques used for the study of the gene function and has thus helped us to gain a better understanding of the many processes through the capture of actively transcribed endogenous genes [21]. By using this method, we previously isolated a new gene, *Mapo1*, which is involved in the O^6^-methylguanine-induced apoptosis [22]. The mouse cell line, KH101, carries a single insertion of the vector sequence in one of alleles of the *Mapo1* gene, and is unable to induce apoptosis properly after treatment with MNU. Therefore, the gene-trapping method is considered to be a powerful tool to identify new genes functioning in the O^6^-methylguanine-induced apoptosis pathway.

By extending the gene-trap mutagenesis screening, we isolated a new gene, *Mapo2*, which is highly conserved among a wide range of multicellular organisms and encodes a protein with characteristic repetitive motifs. This study found that the gene product may be involved in the execution of apoptosis induced by O^6^-methylguanine.

## Results

### Isolation of a mouse cell line defective in the *Mapo2* gene

Retrovirus-mediated gene-trap mutagenesis was performed to identify new genes functioning in the process of apoptosis triggered by O^6^-methylguanine, as described previously [22]. MNU-sensitive mouse-derived cells, due to the defect in MGMT activity, were infected with the gene-trap vector pLHΔU3L-Neo, carrying a promoterless hygromycin B resistance gene, and hygromycin-resistant cells were selected. From Hyg^r^ cells, which carry the vector sequence within actively transcribed genes, MNU-resistant clones were isolated as candidates that were defective in genes related to MNU-induced apoptosis. The genes disrupted in the mutant clones were identified by an inverse PCR, which amplifies sequences spanning the junctions between the genomic DNA and the integrated vector sequences, followed by the determination of the DNA sequences. A database search revealed that one of the mutants had an insertion of the vector in a sequence corresponding to the fifth intron of an uncharacterized gene. The gene LOC78806, encoding 341 amino acids, was located in the D3 locus of mouse chromosome 4, and corresponds to the human gene *C1orf201*, the function of which is not known. The gene is novel, and thus, we named it *MAPO2* for O^6^-methylguanine-induced apoptosis 2.

**Figure 1:**
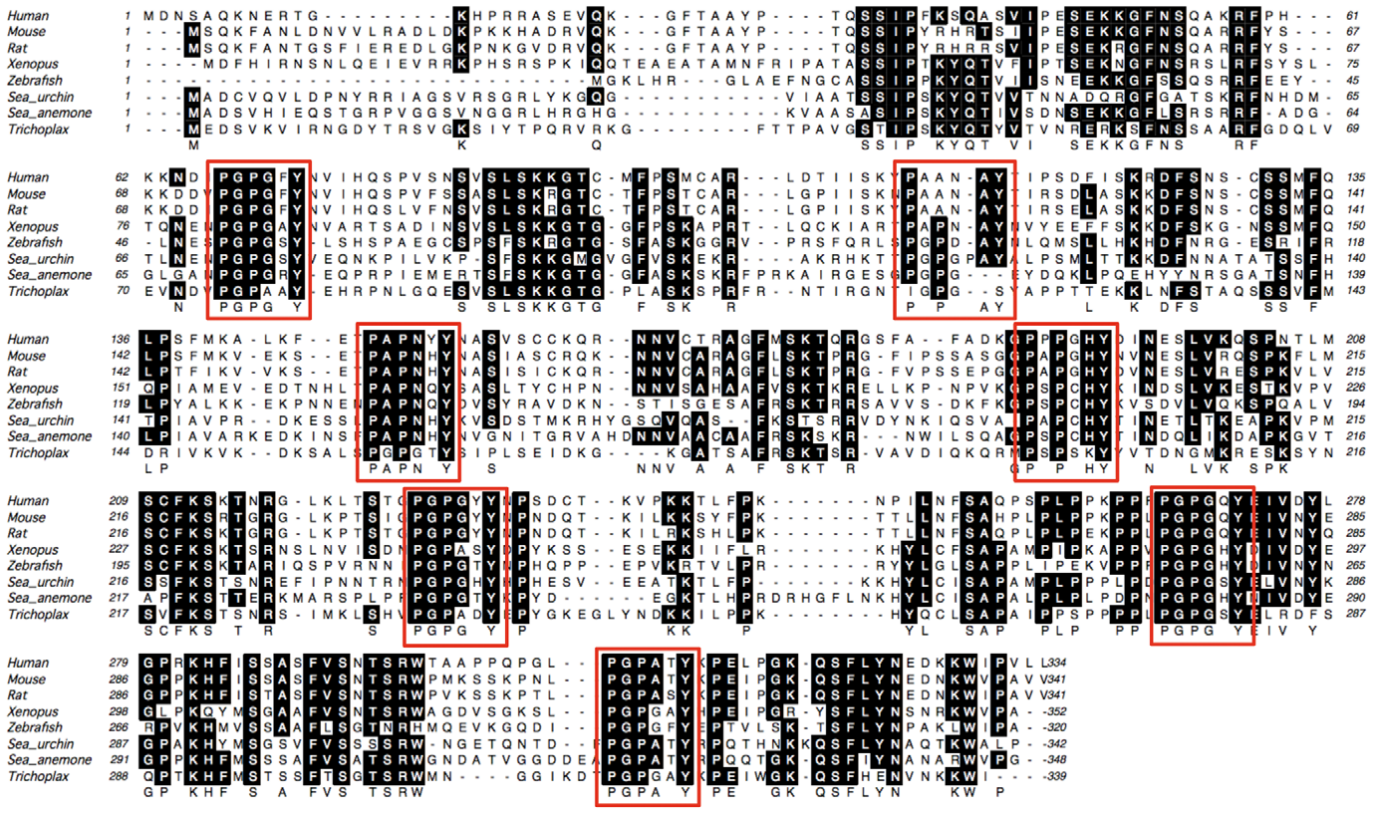
Comparison of the amino acid sequences of MAPO2 proteins in various organisms. Amino acid sequences of MAPO2 homologues in human (*Homo sap*ience: NP_001185941.1), mouse (*Mus musculus*: NP_084465.1), rat (*Rattus norvegicus*: NP_001020943.1), Xenopus (*Xenopus laevis*: NP_001089693.1), Zebrafish (*Danio rerio*: AAH95808.1), sea urchin (*Strongylocentrotus purpuratus*: XP_797702.1), sea anemone (*Nematostella vectensis*: XP_001627297.1), and Trichoplax (*Trichoplax adhaerens*: XP_002114431.1) were aligned using the Clustal W program (http://www.genome.jp/tools/clustalw/). The repetitive PxPxxY motifs are enclosed by squares. Conserved amino acids among more than five sequences are highlighted in white.

A homology search (http://blast.ncbi.nlm.nih.gov/Blast.cgi) revealed that the related amino acid sequences to be present in a wide range of organisms, ranging from humans to some lower multicellular organisms, including *Trichoplax adhaerence*. The prospective proteins carry characteristic 7 PxPxxY repeats, which are evenly distributed along the entire of sequences (**Fig. 1**). The human *MAPO2/C1orf201* is located in the p36.11 locus of chromosome 1. The mouse-derived mutant cells might have mutations not only in the hypothetical gene but also in other unidentified gene, thus the cells are not suitable for further analysis of the effects by the disruption of the hypothetical gene. For the functional analysis of this hypothetical gene, the current study used the human-derived HeLa MR cell line, which is defective for MGMT activity and readily undergoes apoptosis after treatment with MNU.

### Construction of a *MAPO2*-knockdown human cell line

**Figure 2:**
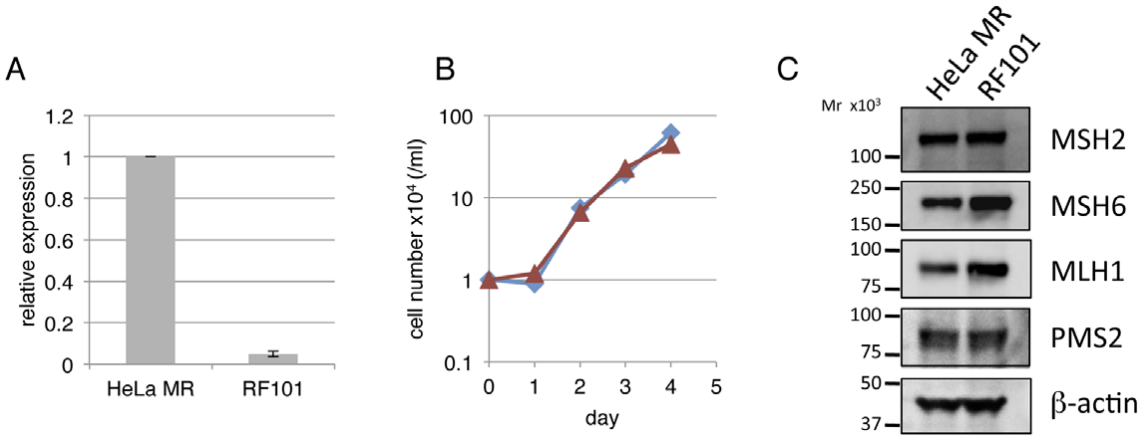
Construction of a HeLa MR-derived *MAPO2*-knockdown cell line. A) The relative expression levels of the *MAPO2* gene in HeLa MR and its derivative RF101 cells, as measured by quantitative real-time RT-PCR. (B) The growth rate of the two types of cells without MNU treatment. The numbers of cells grown under normal conditions were counted every 24 h and plotted. The experiments were performed 3 times, independently, and average values from the 3 experiments were plotted. Diamonds, HeLa MR; triangles, RF101. (C) The levels of MMR proteins. The expression levels of MMR proteins in HeLa MR and RF101 cells were analyzed using immunoblotting with anti-MSH2, anti-MSH6, anti-MLH1 and anti-PMS2 antibodies. β-Actin was used as a loading control.

To analyze the function of the *MAPO2* gene in the induction of apoptosis triggered by MNU, a stable human cell line defective in both *MAPO2* and *MGMT* was constructed. A vector that expressed miRNA specific for *MAPO2* was introduced to HeLa MR cells, which are defective in MGMT expression, and the strain was named RF101. A quantitative real-time PCR analysis showed that the expression level of the *MAPO2* gene in RF101 cells is reduced to about 5% of that of HeLa MR (**Fig. 2A**). The RF101 cells grew at almost same rate as did HeLa MR cells (**Fig. 2B**). The doubling times for HeLa MR and RF101 were both about 16 h, thus suggesting that the function of MAPO2 is not required for cell growth under normal conditions. Moreover, an immunoblotting analysis revealed that *MAPO2*-knockdown does not affect the expression levels of MMR proteins, which are required for the initial step of damage-recognition for MNU-induced apoptosis (**Fig. 2C**).

### Suppression of MNU-induced apoptosis by *MAPO2* knockdown

**Figure 3:**
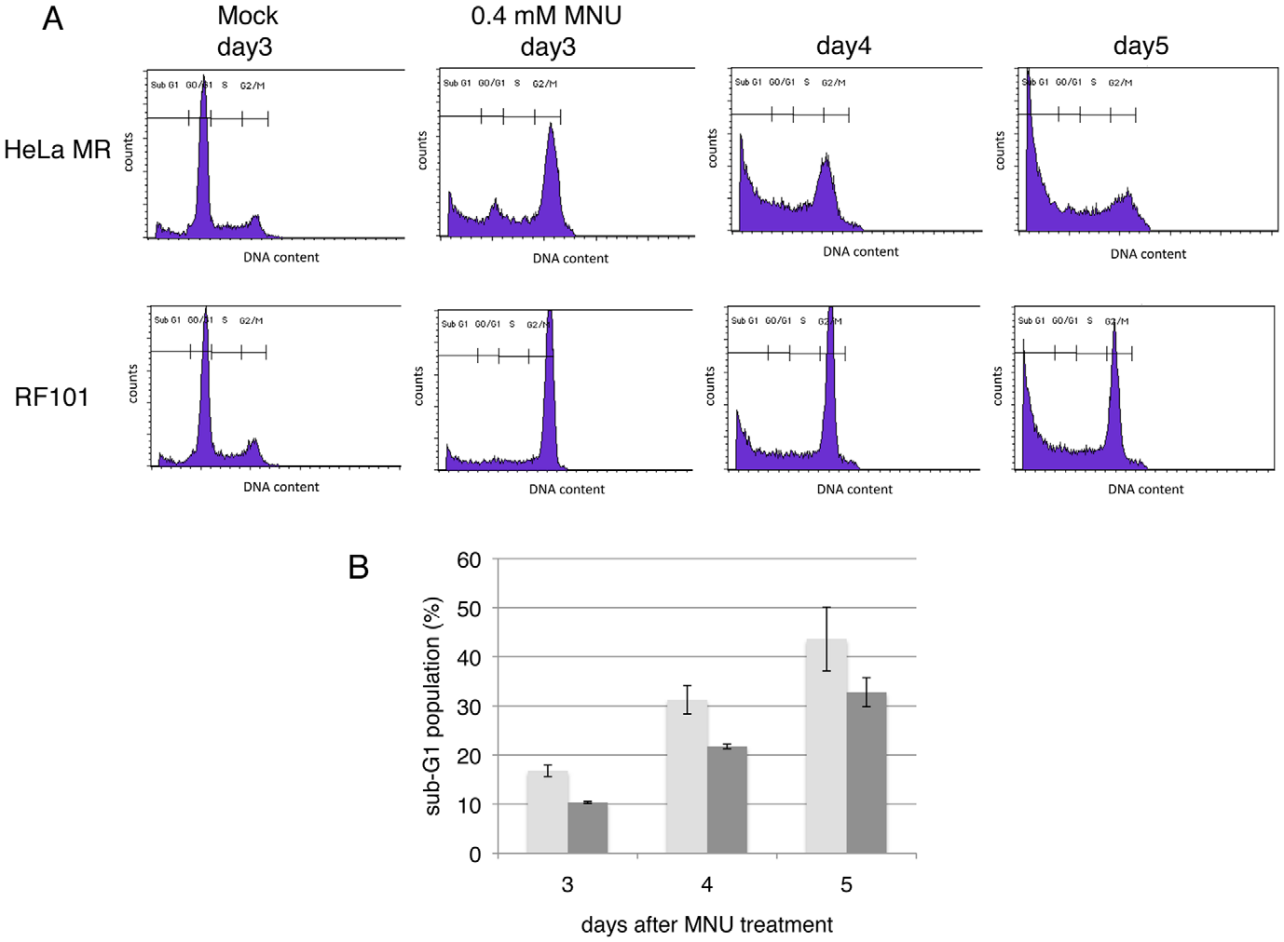
The suppression of apoptosis in *MAPO2*-knockdown cells. (A) The cell cycle profiles of cells after MNU treatment. HeLa MR and RF101 cells were treated with 0.4 mM MNU for 1 h and incubated for 3, 4, and 5 days. The cells were harvested and subjected to a flow cytometric analysis. The representative cell cycle profiles of these cells, treated with or without MNU, are shown. (B) The sub-G_1_ population of cells after MNU treatment. The mean values of sub-G_1_ cell population obtained from more than three independent experiments in (A) and the standard deviations are presented. Light gray bars, HeLa MR; dark gray bars, RF101.

In order to investigate whether the *MAPO2* gene is involved in the MNU-induced apoptosis, HeLa MR and *MAPO2*-knockdown RF101 cells were treated with 0.4 mM MNU and, then were subjected to a flow cytometric analysis after incubation for 3, 4, and 5 days. The cell cycle checkpoint was activated 3 days after MNU treatment, thus resulting in the accumulation of cells at G_2_/M phase (**Fig. 3A**). The sub-G_1_ cell population increased in both HeLa MR and RF101 cells. However, the degree of apoptotic induction in RF101 cells (10%) was significantly lower than that obtained in HeLa MR cells (17%; **Fig. 3A and B**). The suppression of the production of sub-G_1_ cells in RF101 samples was also observed at later time points and about 38% of the cell population in the RF101 still remained at the G_2_/M phase even at day 5, whereas only 19% of the cell population was there in the HeLa MR. This decrease of the sub-G_1_ cell population in RF101 cells after treatment with MNU was not observed when the cells were exposed to a DNA interstrand-cross-linker, 1-(4-amino-2-methyl-5-pyrimidinyl)methyl-3-(2-chloroethyl)-3-nitrosourea (ACNU), and a DNA double-strand break inducer, etoposide (data not shown). These results imply that MAPO2 might be involved in the induction of apoptosis caused by MNU-induced O^6^-methylguanine.

### Effects of *MAPO2*-knockdown on apoptosis-related events

**Figure 4:**
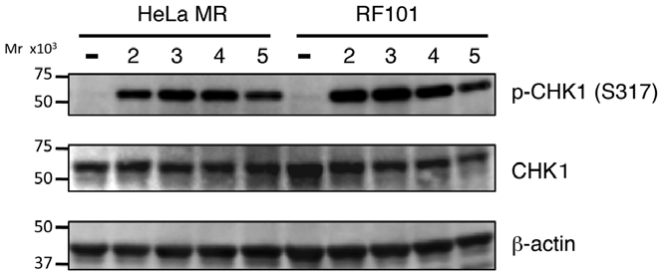
The effect of *MAPO2* knockdown on the cell cycle checkpoint. HeLa MR and RF101 cells were treated with 0.4 mM MNU for 1 h and then collected at indicated times. The whole extracts prepared from the cells were subjected to an immunoblotting analysis to detect the phosphorylated form of CHK1, CHK1 and β-actin, using the respective specific antibodies.

The status of phosphorylation of CHK1, which is a downstream target of ATR, a DNA damage sensor kinase, was analyzed to determine whether cell cycle checkpoint is indeed induced in *MAPO2*-knockdown RF101 cells after MNU treatment. The phosphorylation of S317 of CHK1 was clearly detected by immunoblotting analysis in RF101 as well as HeLa MR cells after treatment with MNU (**Fig. 4**). These data indicated that the function of MAPO2 is dispensable for the activation of the cell cycle checkpoint.

**Figure 5:**
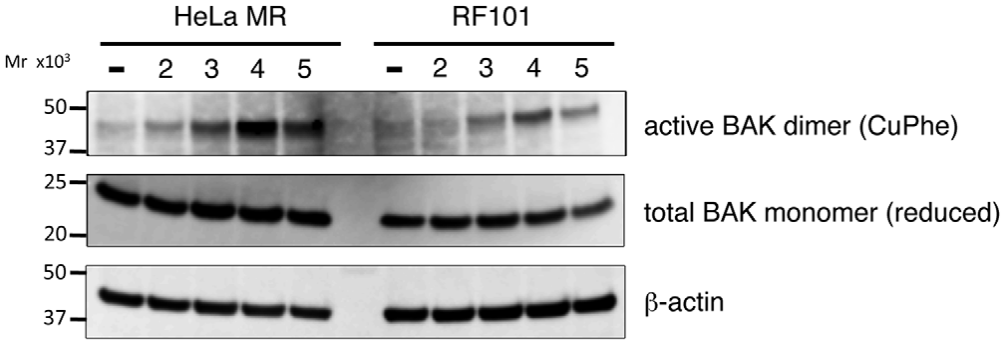
The effect of *MAPO2* knockdown on the activation of BAK proteins. Soluble protein fractions were extracted from HeLa MR and RF101 cells harvested 2, 3, 4 and 5 days after MNU treatment. The extracts were treated with disulphide bonding inducer to form intermolecularly linked active BAK dimers. The samples were boiled with (reduced) or without (CuPhe) 2-mercaptoethanol and subjected onto SDS-PAGE followed by immunoblotting analysis to detect the total amounts of BAK monomers and active BAK dimers, respectively, using anti-BAK monoclonal antibody ab-1. β-Actin was used as a loading control. The molecular weights are shown on the left.

**Figure 6:**
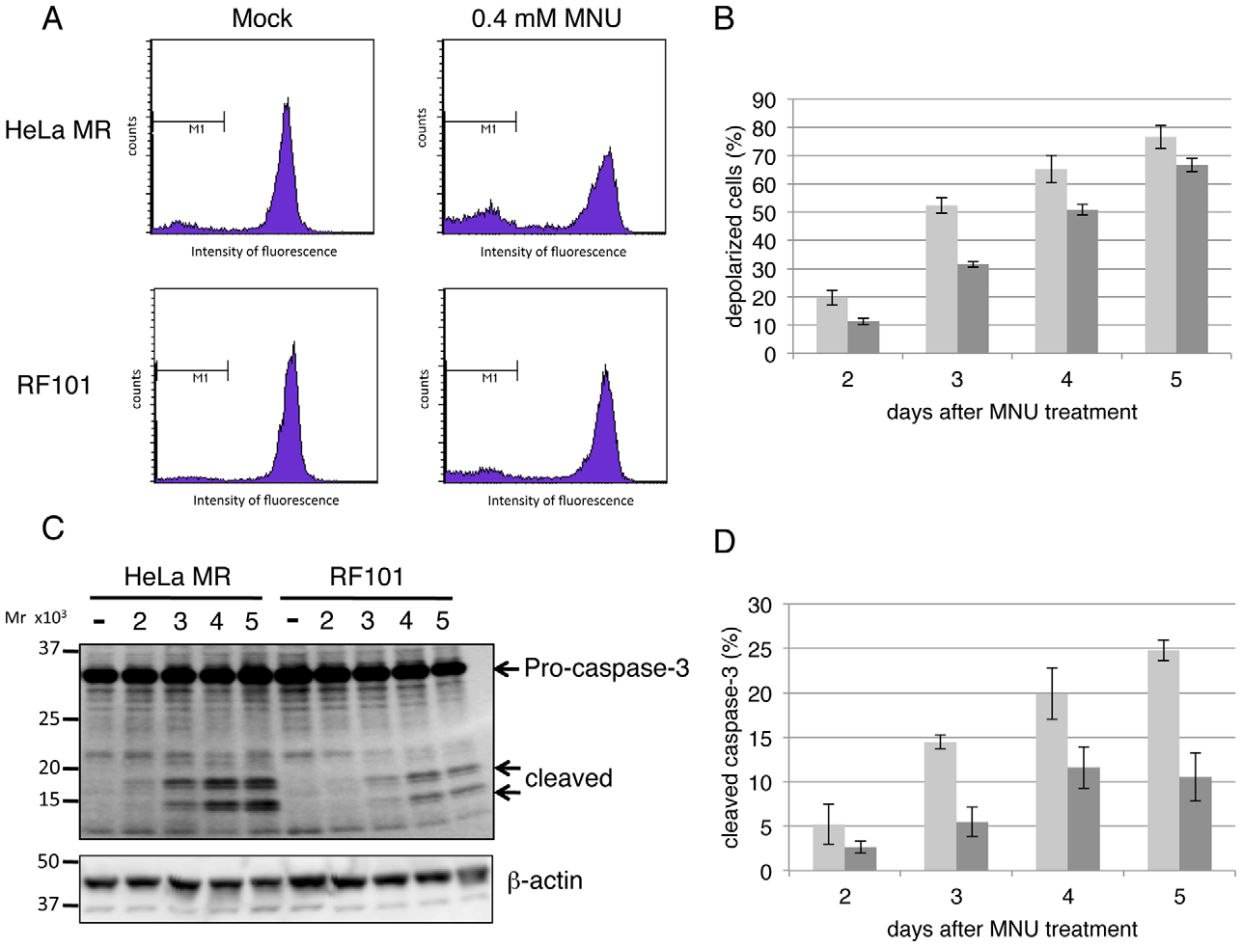
The involvement of MAPO2 in MNU-induced apoptosis. HeLa MR and RF101 cells were treated with or without 0.4 mM MNU for 1 h. (A) Depolarization of the mitochondrial membrane. The cells harvested 2, 3, 4, and 5 days after MNU treatment were subjected to a flow cytometric analysis to monitor mitochondrial membrane depolarization. The representative patterns of the assay at 2 days after Mock- and MNU-treatment are shown. (B) The levels of mitochondrial membrane depolarization. The mean values of mitochondrial membrane depolarized cells at day 2, 3, 4 and 5, and the standard deviations are presented. Light gray bars, HeLa MR; dark gray bars, RF101. (C) The activation of caspase-3. The whole cell extracts prepared from the cells were used for immunoblotting with an anti-caspase-3 antibody, to detect pro-caspase-3 and cleaved caspase-3. (D) The levels of caspase-3 activation. The intensity of the bands, corresponding to pro- and cleaved caspase-3, detected in (C) were measured. The mean values of the ratio of cleaved caspase-3 to total caspase-3 (sum of pro-caspase-3 and cleaved caspase-3), and the standard deviations calculated from four independent experiments are shown. Light gray bars, HeLa MR; dark gray bars, RF101.

BAK, a member of the pro-apoptotic BCL-2 family of proteins, plays a crucial role in the mitochondria-dependent apoptosis pathway [23]. As shown in **Fig. 5**, the activation of BAK was observed in HeLa MR cells during the process of MNU-induced apoptosis, as was evident by the finding that the amount of the active form of BAK homodimer, stabilized by the introduction of disulphide bonding, increased gradually. The total amount of BAK, detected as the monomer form under reducing conditions, was not altered. In *MAPO2*-knockdown RF101 cells, however, the formation of active BAK dimers was significantly inhibited, thus suggesting that the cells were unable to induce apoptosis effectively even after the treatment with MNU. To obtain further evidence supporting that *MAPO2*-knockdown cells are defective in the induction of apoptosis, the effect of *MAPO2*-knockdown was investigated on the depolarization of the mitochondrial membrane and the activation of caspase-3, which are known to occur during the process of apoptosis [20], [24]. The two types of cells were treated with 0.4 mM MNU for 1 h and were then subjected to both assays. In the flow cytometric analysis, the depolarization of mitochondria was gradually induced in HeLa MR (*MAPO2*-positive) cells after treatment with MNU, whereas such depolarization was significantly suppressed in RF101 (*MAPO2*-knockdown) cells, especially at day 2 and 3 (**Fig. 6A and B**). The processing of caspase-3 was examined by immunoblotting and the results were shown in **Fig. 6C and D**. The bands corresponding to cleaved caspase-3 were clearly detected at day 3 after the MNU treatment in HeLa MR cells and the intensity of the bands further increased at day 4 and 5. However, the bands were significantly weaker in RF101 cells and at day 5 the level of cleavage was 40% of that of HeLa MR cells, indicating the inefficient activation of caspase-3 due to the knockdown of the *MAPO2* gene. Taken together, it is strongly suggested that MAPO2 might play an important role in the induction of apoptosis triggered by O^6^-methylguanine.

## Discussion

The gene-tap mutagenesis method was used to isolate mouse-derived mutant cell lines that acquired resistance to MNU even in the absence of MGMT activity. One of the mutant cell lines carried an insertion in the *Mapo2/C1orf201* gene, which had been recognized only by sequence analogies of the human genome. Knocking down of the *MAPO2* gene in human HeLa MR cells, which readily undergo apoptosis after the treatment with MNU, was performed to reveal the function of the *MAPO2* gene in MNU-induced apoptosis. Although the DNA damage-induced cell cycle checkpoint at G_2_/M phase was little affected by the *MAPO2*-knockdown, the production of the sub-G_1_ cell population, the depolarization of the mitochondrial membrane and the activation of BAK and caspase-3, all of which are hallmarks for the induction of apoptosis, were significantly suppressed in the knockdown cells. Thus, we conclude that MAPO2 may be a new gene that is involved in the O^6^-methylguanine-induced apoptosis.

The human *MAPO2* encodes a protein with a calculated molecular mass of 36,786, and the gene is conserved throughout various multicellular organisms. A notable feature of the protein is that the amino acid sequence is rich in proline, which alone accounts for 12.3% of the total number of residues and that it contains evenly distributed 7 PxPxxY repeats, although one of the repeats has Ala instead of Pro at the third residue. A similar repeat is also found in mammalian sperm tail protein, SHIPPO 1, which contains 6 Pro-Gly-Pro repeats in its polypeptides composed of 254 amino acids [25]. The SHIPPO 1 localizes on the sperm flagella outer dense fibers and is likely to associate with different components that characterize the domains of the sperm tail. Proline is an α-helix breaker residue, thus the Pro-Gly-Pro repeats of SHIPPO 1 might play an important role in the final conformation of the protein [25]. Since the predicted secondary structure of MAPO2 is a highly coiled protein, as is observed in SHIPPO 1, it may be inferred that the PxPxxY repeats in the protein may contribute to the formation of its tertiary structure, through which MAPO2 would associate with other apoptosis-related proteins. The identification of the interacting proteins would therefore help to achieve a better understanding of the molecular function of MAPO2 in MNU-induced apoptosis. The subcellular localization of the MAPO2 protein has not been determined to date, because of the unavailability of specific antibodies that recognize the endogenous protein. Therefore, FLAG-tagged mouse MAPO2 was expressed in mouse YT102 cells to determine the localization of the protein. The indirect immunofluorescent analysis using an anti-FLAG antibody showed that mMAPO2 was mainly present in the cytoplasm and also in the nucleus (**Fig. S1**). The FLAG-tagged protein was also detected by immunoblotting analysis in the chromatin-enriched fraction as well as in the cytosolic fraction (data not shown).

The knockdown of the *MAPO2* expression could effectively suppress the induction of apoptosis triggered by MNU. However, the survival fraction of the knockdown cells, as measured by counting the number of colonies formed 10 days after MNU treatment, was comparable to that of *MAPO2*-positive HeLa MR cells, indicating that the knockdown of the *MAPO2* expression does not prevent ultimate cell death (data not shown). There could be at least two possible explanations as follows; i) The small amount of MAPO2 protein expressed under the knockdown condition (about 5% of mRNA level in comparison to the wild-type) would belatedly induce apoptosis and lead to cell death. ii) An alternative cell death pathway, e.g. caspase-independent apoptosis or necrotic cell death, might be employed, which could bypass the dysfunction of the MAPO2-dependent apoptosis pathway. The establishment of *Mapo2*-knockout cells would be worthwhile to gain clues to elucidate the role of the protein in the induction of apoptosis triggered by O^6^-methylguanine.

## Materials and Methods

### Ethics Statement

N/A

### Cell lines and cultivation of cells

Mouse cell lines were established from the lung tissue of mice [17]. Human cell line, HeLa MR, which was originally obtained from H. Hayakawa [26], was used from our laboratory stocks. All the cell lines used are defective in O^6^-methylguanine-DNA methyltransferase activity. The cells were cultivated in Dulbecco's modified Eagle's medium (D-MEM, Wako Pure Chemical Industries Ltd.) containing 10% fetal bovine serum at 37°C in 5% CO_2_.

### Gene-trap mutagenesis and isolation of MNU-resistant clones

Gene-trap mutagenesis was performed as described previously with some modifications [22]. MNU-sensitive mouse-derived cells, due to a defect in MGMT activity, were infected with a modified pLHΔU3L retrovirus vector [22], pLHΔ3L-Neo, carrying a promoterless hygromycin B resistance gene and a neomycin resistance gene under the control of a polII promoter. The cells were sequentially selected in a medium containing 0.3 mg/ml of hygromycin B (Sigma) and in a medium with 0.5 mg/ml of neomycin (Sigma), treated with 0.4 mM MNU for 1 h and further incubated in the drug-free medium. The colonies that formed were isolated as MNU-resistant clones.

### Construction of *MAPO2*-knockdown cell line

A miRNA expression vector, pMAPO2KD1, was constructed using the BLOCK-iT Pol II miR RNAi Expression Vector Kit with EmGFP (Invitrogen, Life Technologies Corp.), according to the manufacturer's instruction manual. The oligonucleotides to express the *MAPO2*-specific miRNA, 5′-TGCTGAGGAGTTTCAAACTTGAGAGCGTTTTGGCCACTGACTGACGCTCTCAATTGAAACTCCT-3′, were purchased from Invitrogen, Life Technologies Corp. HeLa MR cells were transfected with pMAPO2KD1 using Lipofectamine 2000 (Invitrogen, Life Technologies Corp.), and selected in medium containing 5 µg/ml blasticidin (Sigma). The stable transfectants were isolated and a line, in which the *MAPO2* gene expression was knocked down, was obtained and named RF101.

### Quantitative real-time PCR analysis

Total RNA was prepared from RF101 cells using the RNeasy Mini Kit (QIAGEN) and used to synthesize cDNAs by using PrimeScript Reverse Transcriptase (Takara Bio Inc.). Real time PCR was performed with the 7500 Real Time PCR System (Applied Biosystems) using SYBR Premix Ex Taq II (Takara Bio Inc.). The PCR primers for a *MAPO2* gene, 5′-CTTGTGAAGCAGTCGCCAAATACAT-3′ and 5′-CACGATCTCATACTGACCAGGACCT-3′, and for the *GAPDH* gene as a reference, 5′-GCACCGTCAAGGCTGAGAAC-3′ and 5′-ATGGTGGTGAAGACGCCAGT-3′, were purchased from Takara Bio Inc.

### Analyses for apoptosis-related activities

The cells grown on dishes were washed with Dulbecco's phosphate-buffered saline (PBS) and treated with 0.4 mM MNU in a serum-free medium buffered with 0.02 M Hepes-HCl (pH 6.0), at 37°C for 1 h. The cells were further incubated in complete medium for 2, 3, 4 and 5 days and then were harvested for the assays. The preparation of the cell extracts to detect the active BAK dimer was basically followed as described earlier [27]. Briefly, the soluble fraction extracted from the cells with 1% digitonin-containing buffer (20 mM Tris-HCl (pH 7.5), 100 mM sucrose, 2.5 mM MgCl_2_, 100 mM KCl and protein inhibitor cocktail (Roche)) was treated with the redox catalyst copper(II)(1,10-phenanthroline)_3_ on ice for 30 minutes. The reaction was quenched by adding 20 mM of EDTA, and the unreacted SH groups were blocked by 20 mM of N-ethylmaeimide. The samples, with or without 2-mercaptoethanol treatment, were analyzed using SDS-PAGE followed by immunoblotting. The cells were treated using MitoProbe DiOC2 (3) Assay kit (Invitrogen, Life Technologies Corp.) for the mitochondrial membrane depolarization assay, as described previously [20], and analyzed using a FACS Calibur flow cytometer (Becton Dickinson). The cells collected were washed with PBS and resuspended in PBS containing 0.1% Triton X-100, 25 µg/ml propidium iodide and 0.1 mg/ml RNaseA. The samples were analyzed by the FACS Calibur flow cytometer to detect the sub-G_1_ population.

### Immunoblotting

Whole cell extracts were prepared by the direct lyses of cells on culture dishes with 2× SDS polyacrylamide gel electrophoresis sample buffer, and then separated by SDS-PAGE, followed by electrotransfer onto a PVDF membrane (Bio-Rad). Anti-phospho-CHK1 (S317) (Bethyl), anti-CHK1 (Santa Cruz), anti-BAK ab-1 (Millipore), anti-MSH2 (Invitrogen, Life Technologies Corp.), anti-MSH6 (BD Biosciences), anti-PMS2 (BD Biosciences), anti-MLH1 (BD Biosciences), anti-caspase-3 (Cell Signaling) and anti-β-actin (Sigma) were used as primary antibodies.

## Supporting Information

Figure S1 **The localization of FLAG-tagged mMAPO2 protein.** Mouse-derived YT102 (*Mgmt* ^−/−^) cells were transfected with pMAPO2CMV10, in which a p3×FLAG-CMV-10 vector (Sigma) contains mouse *Mapo2* cDNA, using Lipofectamine 2000 (Invitrogen, Life Technologies Corp.). The cells were incubated for 24 h, washed with PBS and fixed in methanol at −20°C for 15 min. Anti-FLAG M2 antibody (Sigma) and Alexa488 conjugated anti-mouse-IgG Goat antibody (Invitrogen, Life Technologies Corp.) were used to detect the FLAG-tagged mMAPO2 protein, and Hoechst33342 (Invitrogen, Life Technologies Corp.) to stain nuclei, for the analyses using fluorescent microscopy. The images for FLAG-tagged mMAPO2, Hoechst33342 and merged signals are represented at the top, middle and bottom, respectively.
(TIF)

## References

[1] Scharer OD, Jiricny J (2001) Recent progress in the biology, chemistry and structural biology of DNA glycosylases. Bioessays 23: 270–281. 11223884 10.1002/1521-1878(200103)23:3<270::AID-BIES1037>3.0.CO;2-J

[2] Seeberg E, Eide L, Bjoras M (1995) The base excision repair pathway. Trends Biochem Sci 20: 391–397. 8533150 10.1016/s0968-0004(00)89086-6

[3] Coulondre C, Miller JH (1977) Genetic studies of the lac repressor. IV. Mutagenic specificity in the lacI gene of Escherichia coli. J Mol Biol 117: 577–606. 416218 10.1016/0022-2836(77)90059-6

[4] Loechler EL, Green CL, Essigmann JM (1984) In vivo mutagenesis by O^6^-methylguanine built into a unique site in a viral genome. Proc Natl Acad Sci USA 81: 6271–6275. 6093094 10.1073/pnas.81.20.6271 PMC391905

[5] Ito T, Nakamura T, Maki H, Sekiguchi M (1994) Roles of transcription and repair in alkylation mutagenesis. Mutat Res 314: 273–285. 7513059 10.1016/0921-8777(94)90071-x

[6] Pegg AE (2000) Repair of O^6^-alkylguanine by alkyltransferases. Mutat Res 462: 83–100. 10767620 10.1016/s1383-5742(00)00017-x

[7] Margison GP, Santibanez-Koref MF (2002) O^6^-alkylguanine-DNA alkyltransferase: role in carcinogenesis and chemotherapy. Bioessays 24: 255–266. 11891762 10.1002/bies.10063

[8] Kaina B, Christmann M, Naumann S, Roos WP (2007) MGMT: key node in the battle against genotoxicity, carcinogenicity and apoptosis induced by alkylating agents. DNA Repair (Amst) 6: 1079–1099. 17485253 10.1016/j.dnarep.2007.03.008

[9] Hickman MJ, Samson LD (1999) Role of DNA mismatch repair and p53 in signaling induction of apoptosis by alkylating agents. Proc Natl Acad Sci USA 96: 10764–10769. 10485900 10.1073/pnas.96.19.10764 PMC17957

[10] Pepponi R, Marra G, Fuggetta MP, Falcinelli S, Pagani E, et al (2003) The effect of O^6^-alkylguanine-DNA alkyltransferase and mismatch repair activities on the sensitivity of human melanoma cells to temozolomide, 1,3-bis(2-chloroethyl)1-nitrosourea, and cisplatin. J Pharmacol Exp Ther 304: 661–668. 12538819 10.1124/jpet.102.043950

[11] Hidaka M, Takagi Y, Takano TY, Sekiguchi M (2005) PCNA-MutSalpha-mediated binding of MutLalpha to replicative DNA with mismatched bases to induce apoptosis in human cells. Nucleic Acids Res 33: 5703–5712. 16204460 10.1093/nar/gki878 PMC1243802

[12] Karran P (2001) Mechanisms of tolerance to DNA damaging therapeutic drugs. Carcinogenesis 22: 1931–1937. 11751422 10.1093/carcin/22.12.1931

[13] Glassner BJ, Weeda G, Allan JM, Broekhof JL, Carls NH, et al (1999) DNA repair methyltransferase (Mgmt) knockout mice are sensitive to the lethal effects of chemotherapeutic alkylating agents. Mutagenesis 14: 339–347. 10375003 10.1093/mutage/14.3.339

[14] Sakumi K, Shiraishi A, Shimizu S, Tsuzuki T, Ishikawa T, et al (1997) Methylnitrosourea-induced tumorigenesis in MGMT gene knockout mice. Cancer Res 57: 2415–2418. 9192819

[15] Tsuzuki T, Sakumi K, Shiraishi A, Kawate H, Igarashi H, et al (1996) Targeted disruption of the DNA repair methyltransferase gene renders mice hypersensitive to alkylating agent. Carcinogenesis 17: 1215–1220. 8681434 10.1093/carcin/17.6.1215

[16] Kawate H, Sakumi K, Tsuzuki T, Nakatsuru Y, Ishikawa T, et al (1998) Separation of killing and tumorigenic effects of an alkylating agent in mice defective in two of the DNA repair genes. Proc Natl Acad Sci USA 95: 5116–5120. 9560238 10.1073/pnas.95.9.5116 PMC20223

[17] Takagi Y, Takahashi M, Sanada M, Ito R, Yamaizumi M, et al (2003) Roles of MGMT and MLH1 proteins in alkylation-induced apoptosis and mutagenesis. DNA Repair (Amst) 2: 1135–1146. 13679151 10.1016/s1568-7864(03)00134-4

[18] Yoshioka K, Yoshioka Y, Hsieh P (2006) ATR kinase activation mediated by MutSalpha and MutLalpha in response to cytotoxic O^6^-methylguanine adducts. Mol Cell 22: 501–510. 16713580 10.1016/j.molcel.2006.04.023 PMC2423943

[19] Ochs K, Kaina B (2000) Apoptosis induced by DNA damage O^6^-methylguanine is Bcl-2 and caspase-9/3 regulated and Fas/caspase-8 independent. Cancer Res 60: 5815–5824. 11059778

[20] Takagi Y, Hidaka M, Sanada M, Yoshida H, Sekiguchi M (2008) Different initial steps of apoptosis induced by two types of antineoplastic drugs. Biochem Pharmacol 76: 303–311. 18573489 10.1016/j.bcp.2008.05.008

[21] Stanford WL, Cohn JB, Cordes SP (2001) Gene-trap mutagenesis: past, present and beyond. Nat Rev Genet 2: 756–768. 11584292 10.1038/35093548

[22] Komori K, Takagi Y, Sanada M, Lim TH, Nakatsu Y, et al (2009) A novel protein, MAPO1, that functions in apoptosis triggered by O^6^-methylguanine mispair in DNA. Oncogene 28: 1142–1150. 19137017 10.1038/onc.2008.462

[23] Wei MC, Lindsten T, Mootha VK, Weiler S, Gross A, et al (2000) tBID, a membrane-targeted death ligand, oligomerizes BAK to release cytochrome c. Genes Dev 14: 2060–2071. 10950869 PMC316859

[24] Cossarizza A, Kalashnikova G, Grassilli E, Chiappelli F, Salvioli S, et al (1994) Mitochondrial modifications during rat thymocyte apoptosis: a study at the single cell level. Exp Cell Res 214: 323–330. 8082735 10.1006/excr.1994.1264

[25] Egydio de Carvalho C, Tanaka H, Iguchi N, Ventela S, Nojima H, et al (2002) Molecular cloning and characterization of a complementary DNA encoding sperm tail protein SHIPPO 1. Biol Reprod 66: 785–795. 11870087 10.1095/biolreprod66.3.785

[26] Hayakawa H, Koike G, Sekiguchi M (1990) Expression and cloning of complementary DNA for a human enzyme that repairs O^6^-methylguanine in DNA. J Mol Biol 213: 739–747. 2359121 10.1016/S0022-2836(05)80260-8

[27] Dewson G, Kratina T, Sim HW, Puthalakath H, Adams JM, et al (2008) To trigger apoptosis, Bak exposes its BH3 domain and homodimerizes via BH3:groove interactions. Mol Cell 30: 369–380. 18471982 10.1016/j.molcel.2008.04.005

