# Supplementary figures and images for: The Identification of a Novel Gene, MAPO2, That Is Involved in the Induction of Apoptosis Triggered by O6-Methylguanine
Source: PLoS One. 2012 Sep 24;7(9):e44817. doi: 10.1371/journal.pone.0044817 (PMC3454368; doi:10.1371/journal.pone.0044817)

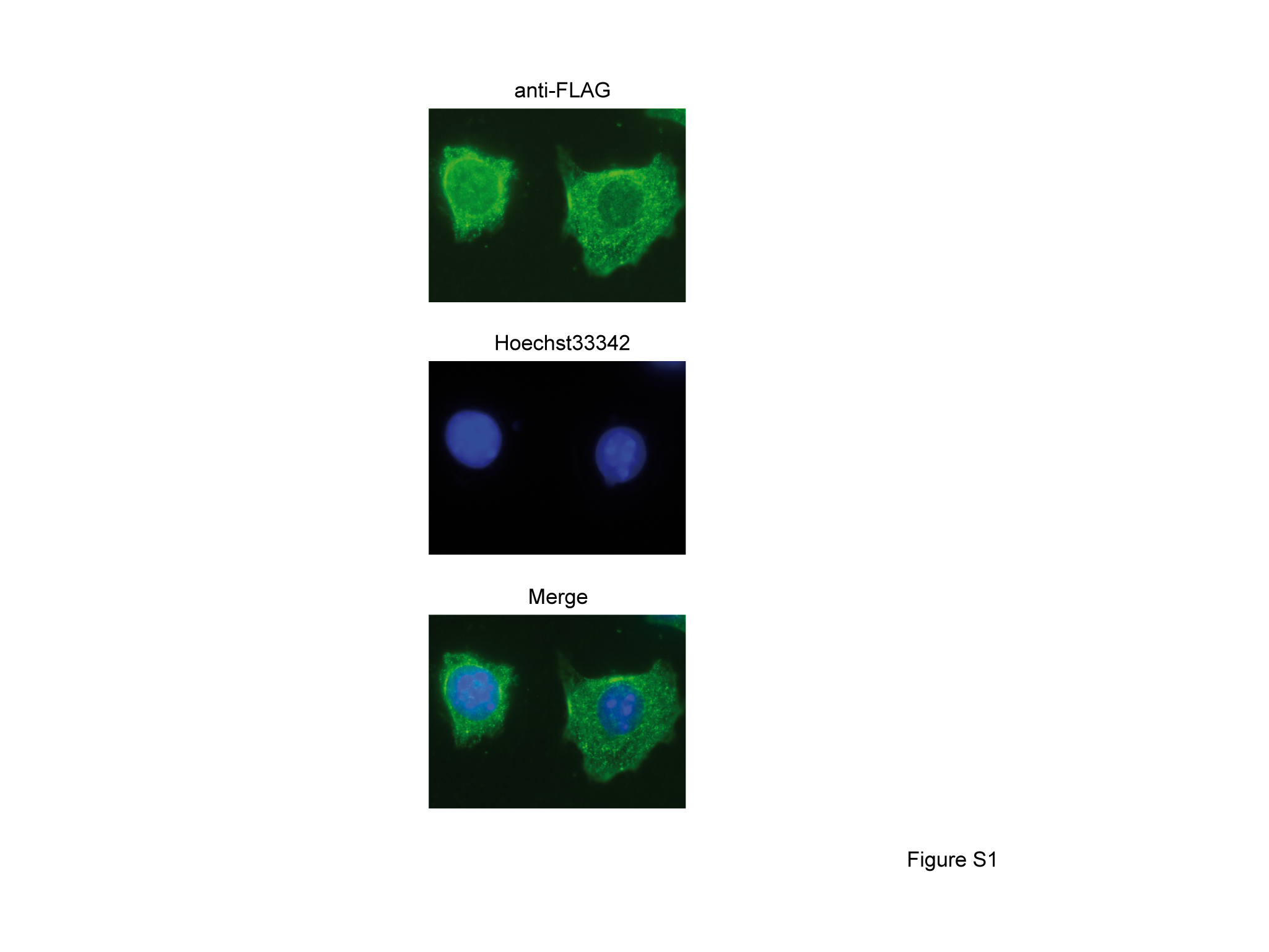

Supplement: Figure S1 — The localization of FLAG-tagged mMAPO2 protein. Mouse-derived YT102 (Mgmt −/−) cells were transfected with pMAPO2CMV10, in which a p3×FLAG-CMV-10 vector (Sigma) contains mouse Mapo2 cDNA, using Lipofectamine 2000 (Invitrogen, Life Technologies Corp.). The cells were incubated for 24 h, washed with PBS and fixed in methanol at −20°C for 15 min. Anti-FLAG M2 antibody (Sigma) and Alexa488 conjugated anti-mouse-IgG Goat antibody (Invitrogen, Life Technologies Corp.) were used to detect the FLAG-tagged mMAPO2 protein, and Hoechst33342 (Invitrogen, Life Technologies Corp.) to stain nuclei, for the analyses using fluorescent microscopy. The images for FLAG-tagged mMAPO2, Hoechst33342 and merged signals are represented at the top, middle and bottom, respectively. (TIF) [file pone.0044817.s001.tif]
